# Timing selection for loosened tooth fixation based on degree of alveolar bone resorption: a finite element analysis

**DOI:** 10.1186/s12903-022-02375-z

**Published:** 2022-08-08

**Authors:** Zhang-yan Ye, Hao Ye, Xi-xi Yu, Yong Wang, Li-jun Wu, Xi Ding

**Affiliations:** 1 Department of Stomatology, Pingyang Hospital Affiliated to Wenzhou Medical University, Wenzhou, 325400 Zhejiang People’s Republic of China; 2 Institute of Digitized Medicine and Intelligent Technology, Wenzhou Medical University, Wenzhou, 325015 Zhejiang People’s Republic of China; 3 Wenzhou Medical University, Wenzhou, 325015 Zhejiang People’s Republic of China; 4 Department of Stomatology, The First Affiliated Hospital of Wenzhou Medical University, Wenzhou, 325015 Zhejiang People’s Republic of China

**Keywords:** Periodontitis, Three-dimensional finite element, Biomechanics, Bone resorption, Loosened tooth fixation

## Abstract

**Objective:**

This study aimed to evaluate timing of fixation to retard bone absorption using finite element analysis(FEA).

**Methods:**

Volunteer CT images were used to construct four models of mandibles with varying degrees of alveolar bone resorption. By simulating occlusal force loading, biomechanical analysis was made on the periodontal membrane, tooth root and surrounding bone (both cancellous and cortical) of mandibular dentition.

**Results:**

The von Mises stress value of the periodontal structures was positively related with the degree of alveolar bone resorption, and the von Mises stress at the interface between the periodontal membrane and tooth root was increased significantly in moderate to severe periodontitis models. The von Mises stress at the interface between the periodontal cortical bone and cancellous bone was increased significantly in the severe periodontitis model. And the von Mises stress value with oblique loading showed significantly higher than vertical loading.

**Conclusion:**

Teeth with moderate to severe periodontitis, loosened tooth fixation can be used to retard bone absorption.

## Introduction

Periodontitis has become an oral disease with very high incidence in China, and has generally, become the leading cause of tooth loss in adults [1]. Clinically, tooth loss caused by chronic periodontitis is often accompanied by severe soft and hard tissue defects, which affects appearance and function, and also brings certain difficulties to denture restoration and implantation [2]. Periodontal disease treatment is mainly synthetic serial treatment, with periodontal drug treatment and surgical treatment to control inflammation, with restoration treatment to adjust occlusion, eliminate tooth trauma and reduce the burden of periodontal supporting tissues. With loosened tooth fixation to disperse occlusal force, control pathological loosening and displacement, and improve chewing efficiency, the affected tooth can get physiological rest, which is beneficial to the control of periodontal inflammation and healing of damaged periodontal tissue, and the oral comfort of patient was improved [3]. After perfect periodontal treatment, tooth looseness of patients with moderate to severe periodontitis can be significantly improved [4].

Due to limitations of medical ethics, it is very difficult to directly measure the intraoral biomechanical characteristics of patients with periodontitis. FEA can be used to make stress analysis of the oral structure, shape, load and mechanical properties of materials for objective, accurate and true stress distribution [5]. Baghdadi D used FEA to explain the biomechanical behaviour of crowded lower front teeth in reduced Periodontium [6]. Many in vitro studies have proved splinting was effective in slowing bone resorption and have measured tooth mobility generated by different dental splint materials to determine their flexibility using FEA [7–9]. However, these studies did not indicate which timepoint of splinting was more benefical. Hence, the aim of this study was to provide a scientific biomechanical basis for the timing of fixation of loosened tooth.

## Materials and methods

### The volunteer

The experimental volunteer was selected from the 2nd generation of Chinese virtual person (living digital-human) "No.23" volunteer, with complete dentition.

### Finite element models

**Fig. 1:**
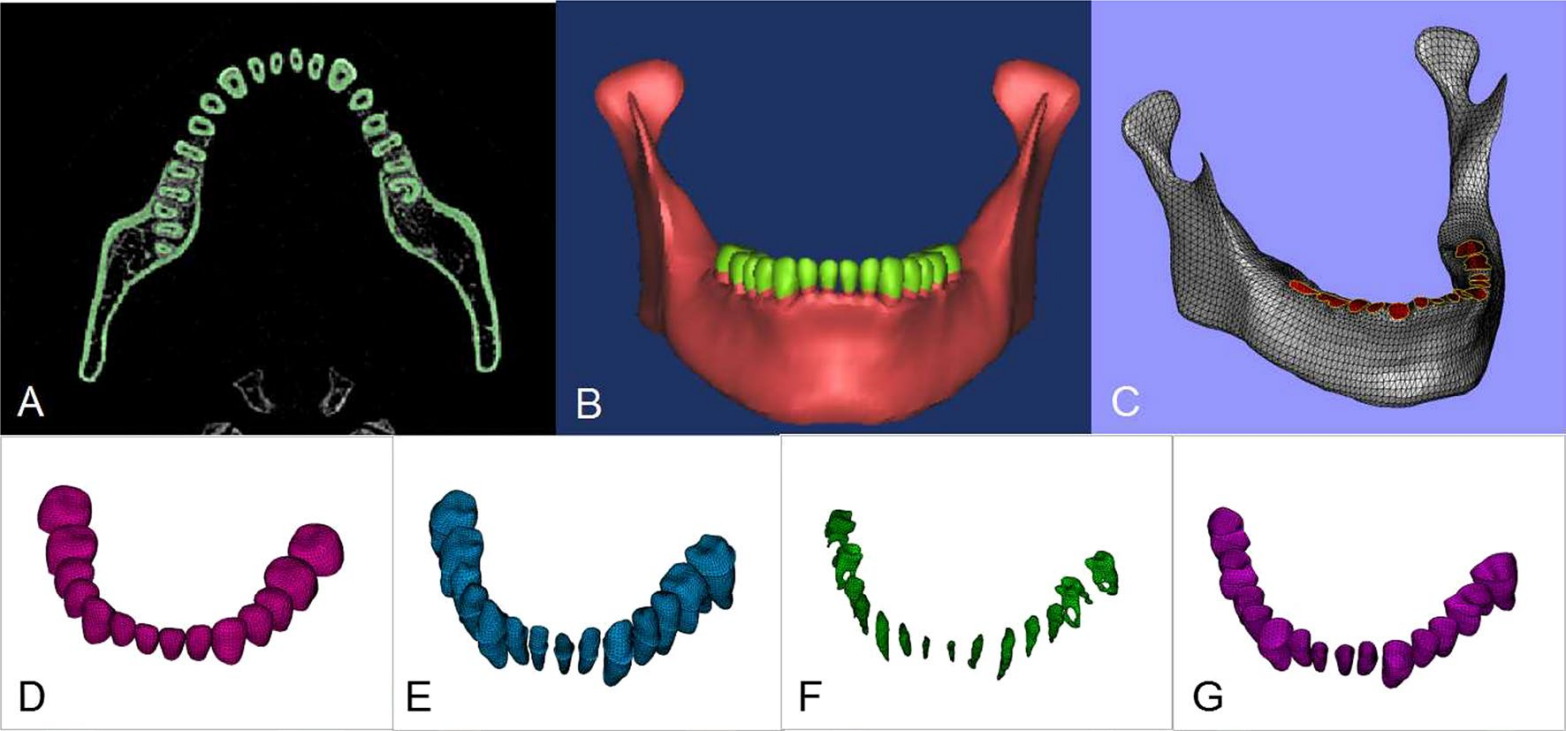
Modeling Steps: **A** CBCT image of living digital-human No. 23. **B** the three-dimensional digital anatomy model of mandible. **C** The mesh refinement of mandible. **D** The mesh refinement of enamel. **E** The mesh refinement of dentin-cementum complex. **F** The mesh refinement of dental pulp. **G** The mesh refinement of periodontal membrane

CT image data of mandible of the volunteer was imported into the medical three-dimensional image reconstruction software Materialise Mimics 10.0 (Materialise Company, Belgium) in DICOM format to generate the bone tissue surface profile. The gap between the tooth root and mandible was filled with a 0.2 mm periodontal membrane structure, and in combination with various software tools and clinical anatomical features, the profile curve of each structure was manually plotted. The three-dimensional digital anatomy model of mandible was reconstructed (including the separation of cortical bone and cancellous bone, enamel, dentin-cementum complex, periodontal membrane, dental pulp, temporomandibular joint condyle and other structures), then imported into reverse engineering software Geomagic Studio12.0 (Geomagic Company, USA) in STL format, using polygon editing tools to optimize the mesh of each independent anatomical structure. Alveolar bone resorption in chronic periodontitis is generally described by the ratio of resorption area to the length of tooth root, and usually divided into three degrees. In order to unify bone resorption of each tooth position in the sample models, the critical value of bone resorption in varying degrees of periodontitis was used as the standard for division. The model was trimmed, and the total length of the measured tooth root was subtracted from the distance of enamel-cementum boundary and alveolar ridge to calculate the root length of the inner alveolar bone of each tooth. The height of alveolar bone was reduced to 0, 1/3, 1/2 and 2/3 of the inner length of the root bone. The edge was trimmed to make it smooth and continuous. Four models were established to simulate (a) non-periodontitis (b) mild periodontitis (c) moderate periodontitis and (d) severe periodontitis of the mandible models, and to simulate the stable state of bone resorption after periodontal treatment. The model was imported into the finite element pre-processing software HyperMesh 11.0 (Altair Company, USA) for materialization. After the Boolean operation, finite element models of a mandible with varying degrees of alveolar bone resorption were constructed (Fig. 1).

### Mesh independency analysis

**Fig. 2:**
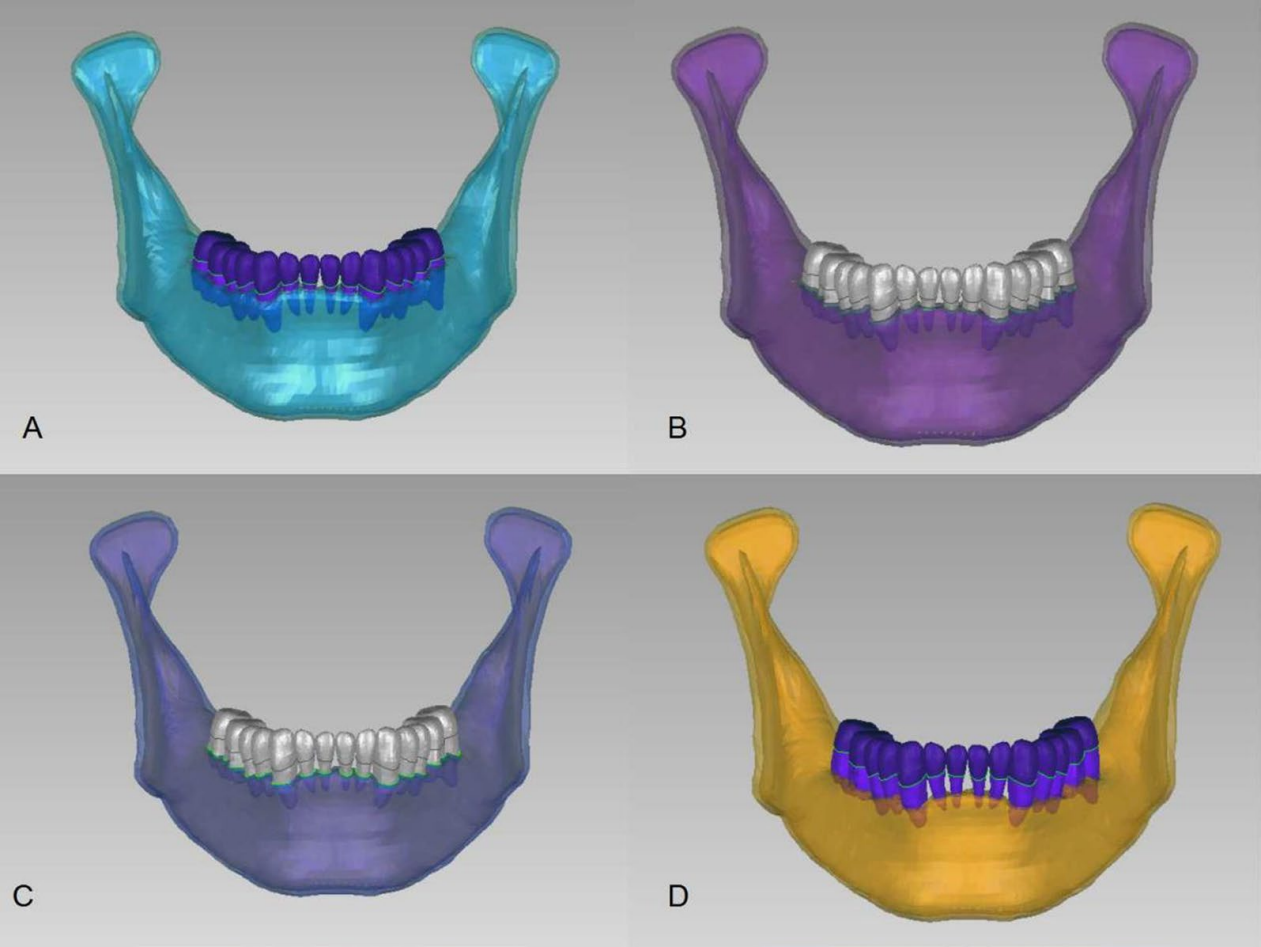
Perspective view of finite element models of mandibles with varying degrees of periodontitis. *Note* In all the charts in this study, **A** normal mandible; **B** mandible with mild periodontitis; **C** mandible with moderate periodontitis; **D** mandible with severe periodontitis

Take the non-periodontitis model vertical loading as an example. The initial mesh size of teeth and surrounding tissues adopted 0.5 mm, and the maximum stress error percentage between 0.5 mm and 0.25 mm was 1.4% (Table 1). Given the calculation efficiency, the four modles (Fig. 2) were established using 0.5 mm mesh size. There were 283,886 tetrahedrons and 53,718 nodes in the non-periodontitis model, 274,913 tetrahedrons and 52,382 nodes in the mild periodontitis model, 273,965 tetrahedrons and 52,719 nodes in the moderate periodontitis model, and 261,095 tetrahedrons and 50,911 nodes in the severe periodontitis model.

**Table 1:** Mesh independency analysis

Mesh size	1 mm	0.5 mm	0.25 mm
Number of element	118,551	283,886	866,193
Max stress (Mpa)	13.88	14.62	14.41

### Experimental preset conditions

All materials were considered linear-elastic, isotropic and homogeneous [10–16]. The applied material properties (elastic modulus and Poisson's ratio) were obtained from the literature (Table 2).

**Table 2:** Material mechanical parameters

Material	Elasticity modulus (Gpa)	Poisson’s ratio	References
Periodontal membrane	0.069	0.450	[10–16]
Cortical bone	13.700	0.300	[11–16]
Cancellous bone	1.370	0.300	[11, 12, 14–16]
Dentin cementum complex	18.600	0.310	[10–12, 14–16]
Enamel	41.000	0.300	[11, 14–16]
Root canal pulp	0.020	0.450	[11, 14–16]

### Loading force

**Fig. 3:**
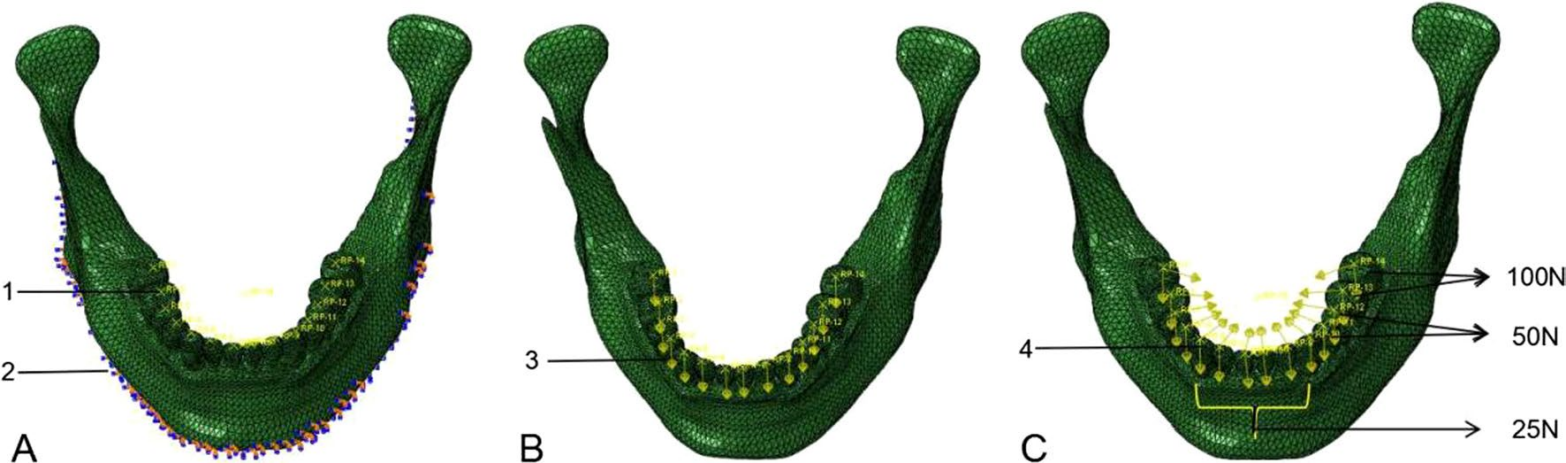
**A** 1. The point of occlusal loading, 2. The fixed edge. **B** 3. Vertical loading. **C** 4. Oblique loading

Different teeth play diverse roles in chewing function, same loading was applied to the teeth of same region. Therefore, the loading of 100 N, 50 N and 25 N were loaded on the occlusal surfaces of molars, premolars, and anterior teeth, respectively [10]. In this study, a certain distributed point on the occlusal surface of each tooth was selected for concentrate loading [17], and the chewing cycle was simplified to vertical loading and oblique loading. Under loading conditions, the edge of the mandible was fixed to prevent the jaw from displacement in the X, Y and Z directions [13, 15] (Fig. 3).

## Results

### Biomechanical analysis of tooth root interface

**Fig. 4:**
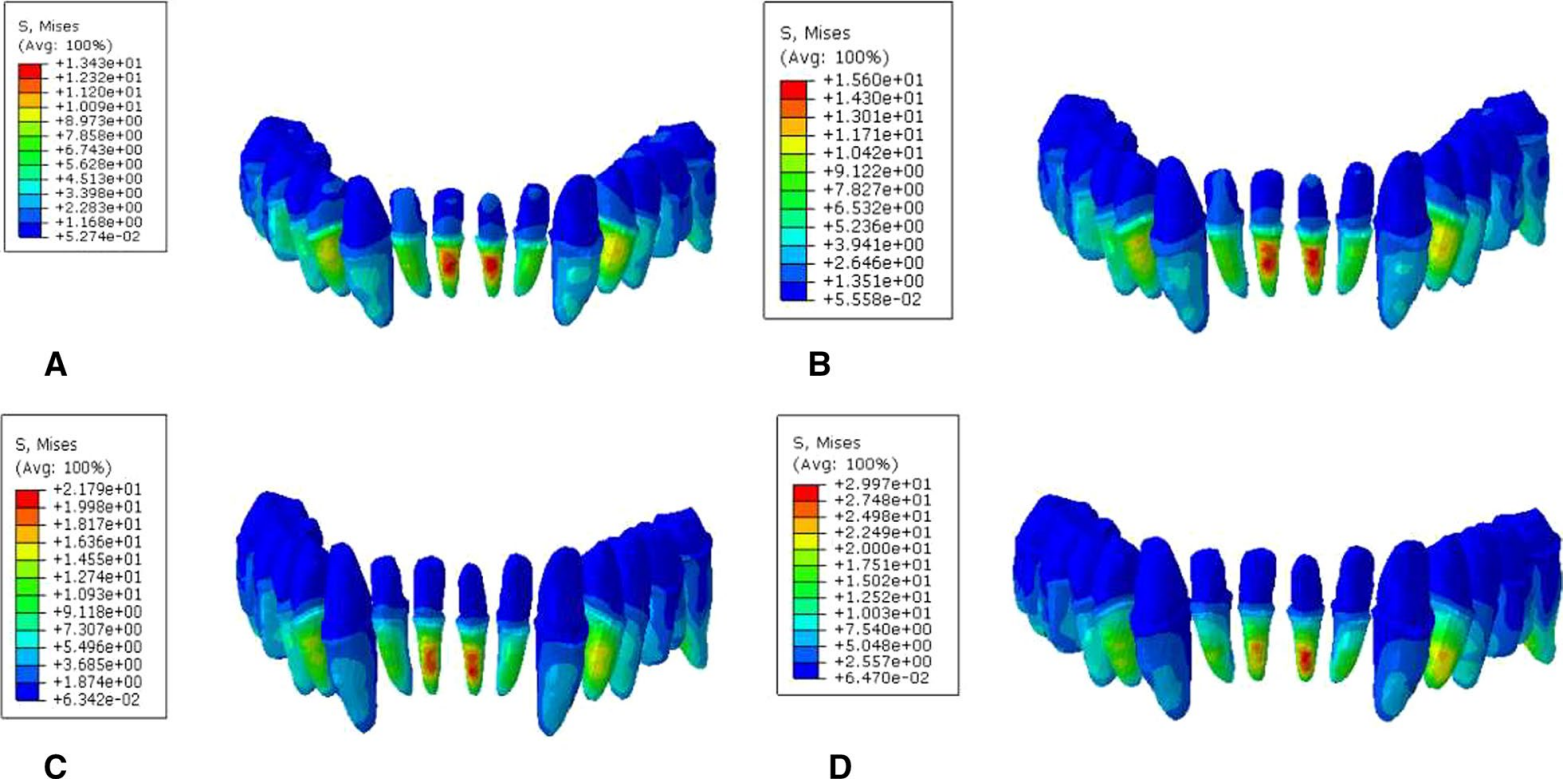
Cloud chart of a finite element analysis of mandibular root interface during vertical loading. **A** normal mandible; **B** mandible with mild periodontitis; **C** mandible with moderate periodontitis; **D** mandible with severe periodontitis

**Fig. 5:**
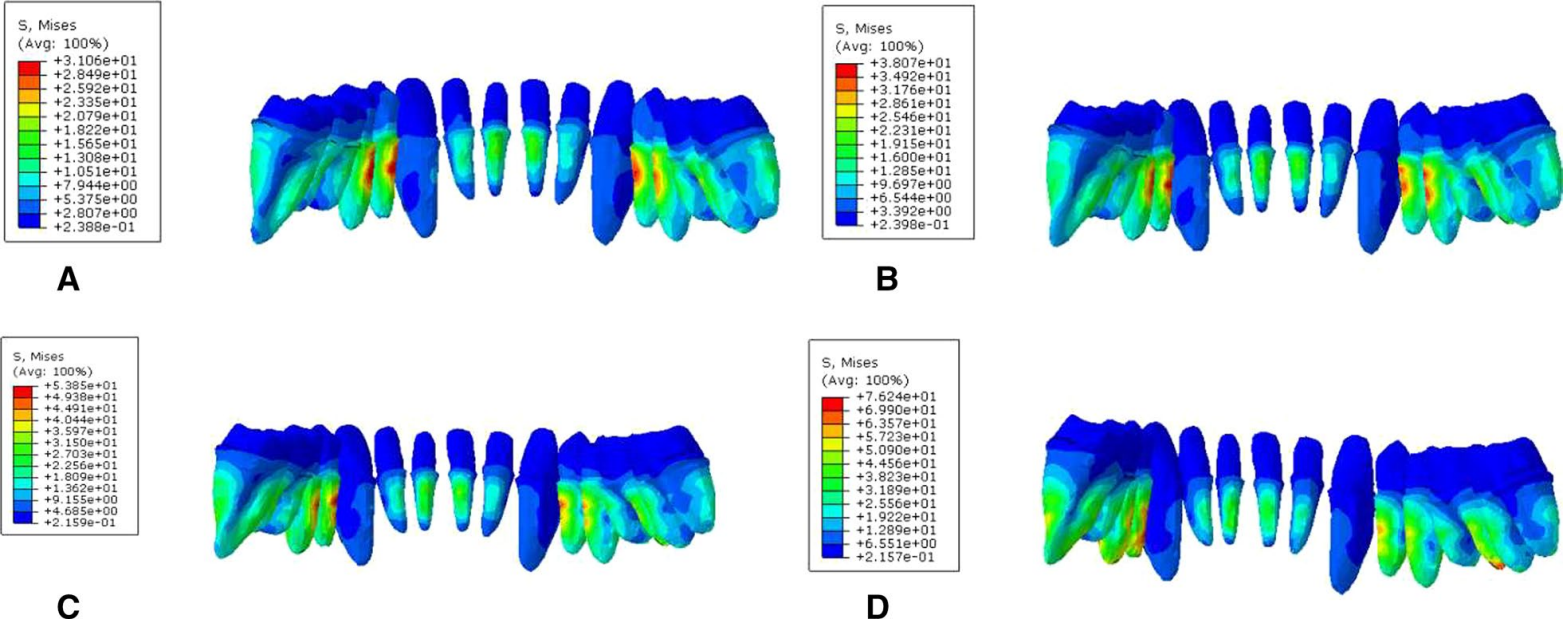
Cloud chart of a finite element analysis of mandibular root interface during 45° oblique loading. **A** normal mandible; **B** mandible with mild periodontitis; **C** mandible with moderate periodontitis; **D** mandible with severe periodontitis

During vertical loading, the von Mises stress distribution on the root surface was concentrated on the root labial side of the lower anterior teeth. The maximum Von Mises stress (MVMS) of mandibular dentition was concentrated on the incisor. The MVMS of the root interface of normal mandible was 13.43 MPa; the MVMS of the root interface of mild periodontitis was 15.60 MPa; the MVMS of the root interface of moderate periodontitis was 21.79 MPa; the MVMS of the root interface of severe periodontitis was 29.97 MPa (Fig. 4, Table 3). During 45° oblique loading, the stress was concentrated on the root surface of mesiolingual posterior teeth, and the MVMS was concentrated on the root surface of the mandibular first molar. The stress was 2–3 times than when vertical loading (Fig. 5, Table 4). In addition, during both vertical and oblique loading, the MVMS was increased with aggravation of periodontitis, and stress was increased more significantly with moderate to severe periodontitis.

**Table 3:** Peak stress of root interface during vertical loading (MPa)

Group	47	46	45	44	43	42	41	31	32	33	34	35	36	37
A	9.24	6.60	5.65	9.88	3.97	8.23	13.04	13.43	7.88	3.82	10.24	5.49	6.56	7.18
B	10.59	7.07	6.94	12.84	4.42	9.12	15.60	15.06	8.794	4.21	11.96	6.28	8.62	8.783
C	13.52	8.21	8.05	14.48	5.44	12.89	20.19	21.79	11.14	5.49	16.66	7.29	10.24	13.83
D	17.39	9.35	9.92	20.30	7.38	18.91	25.55	29.97	16.91	7.09	24.05	9.94	11.73	20.84

**Table 4:** Peak stress of root interface during 45° oblique loading (MPa)

Group	47	46	45	44	43	42	41	31	32	33	34	35	36	37
A	20.66	25.68	27.19	29.14	9.69	6.08	17.69	18.58	7.693	12.25	30.96	31.06	18.78	14.91
B	23.54	30.03	36.43	34.63	6.85	11.96	21.32	21.87	14.82	7.30	38.07	36.34	25.48	18.86
C	32.30	43.32	47.13	48.65	8.38	18.07	34.02	31.68	19.65	9.72	53.85	50.56	33.25	31.97
D	45.33	76.24	54.70	63.88	12.35	29.71	37.76	42.45	31.86	12.70	73.17	69.41	53.7	57.21

### Biomechanical analysis of periodontal membrane interface

**Fig. 6:**
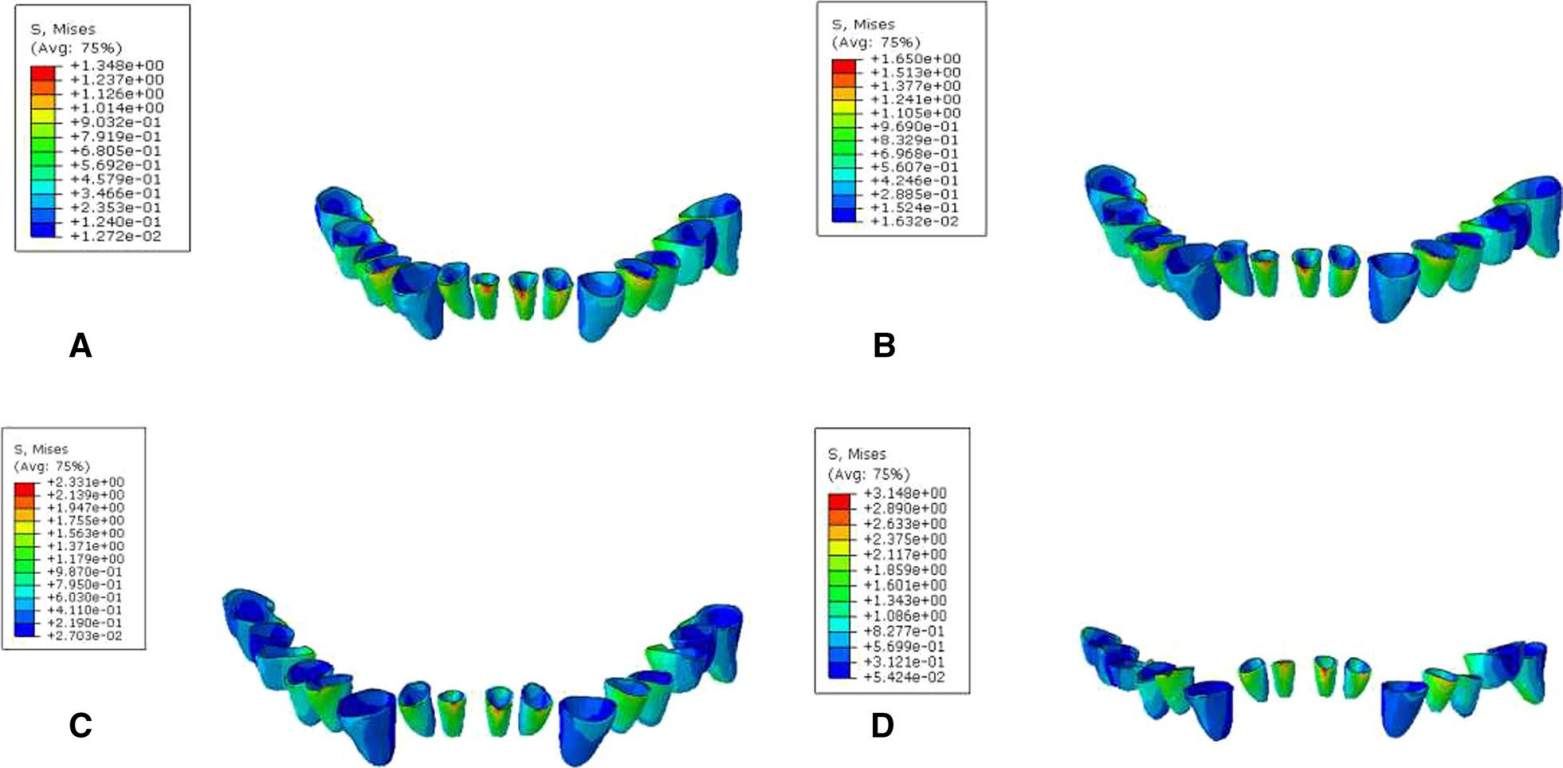
Cloud chart of a finite element analysis of a periodontal membrane of mandibular dentition during vertical loading. **A** normal mandible; **B** mandible with mild periodontitis; **C** mandible with moderate periodontitis; **D** mandible with severe periodontitis

**Fig. 7:**
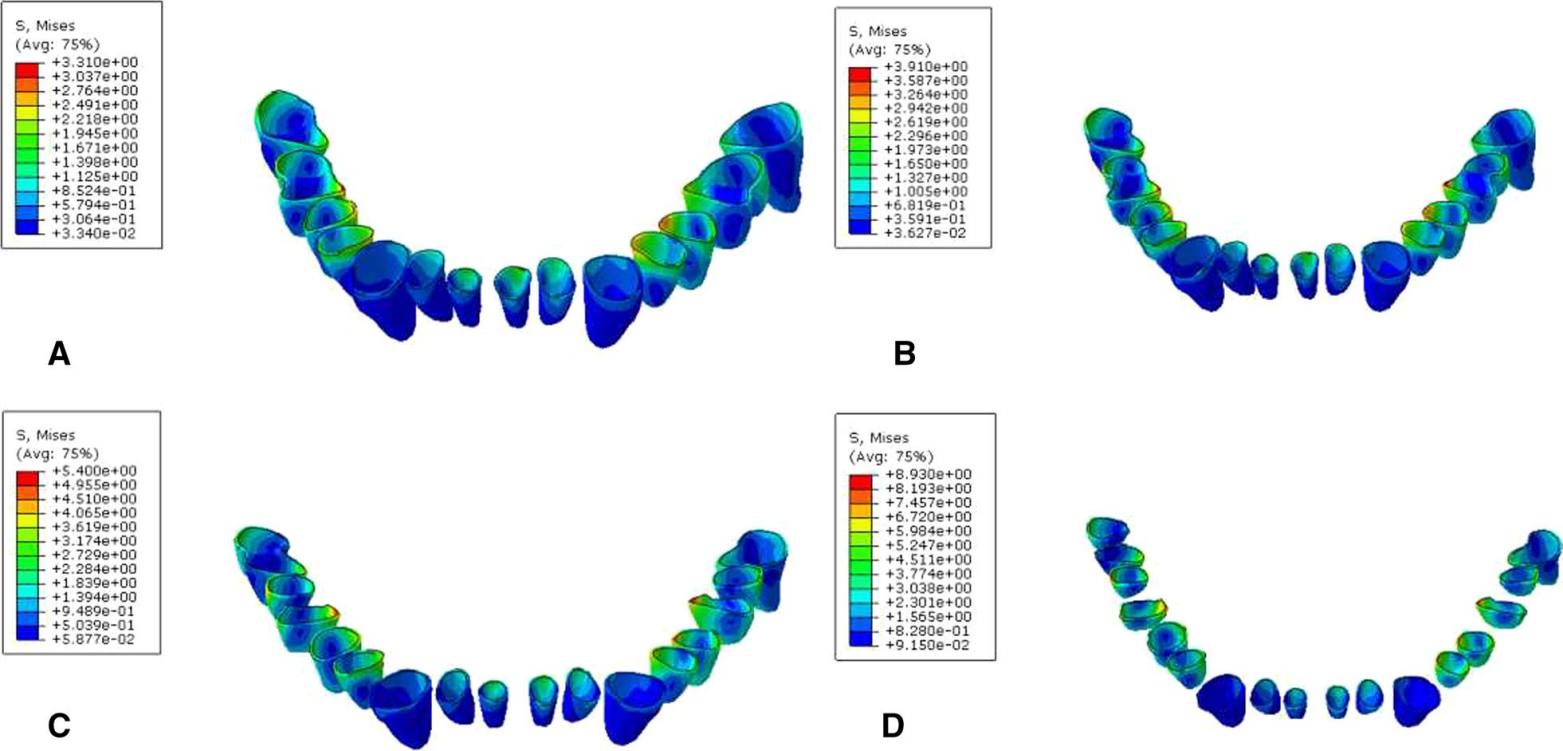
cloud chart of a finite element analysis of a periodontal membrane of mandibular dentition during 45° oblique loading. **A** normal mandible; **B** mandible with mild periodontitis; **C** mandible with moderate periodontitis; **D** mandible with severe periodontitis

During vertical loading, the von Mises stress distribution of the periodontal membrane was concentrated at the corresponding position of periodontal membrane on the labial side of the lower anterior tooth root close to the neck. The MVMS was concentrated in the incisor. The MVMS of the periodontal membrane of a normal mandibular was 1.35 MPa; the MVMS of the periodontal membrane with mild periodontitis was 1.65 MPa; the MVMS of the periodontal membrane with moderate periodontitis was 2.33 MPa; the MVMS of the periodontal membrane with severe periodontitis was 3.15 MPa (Fig. 6, Table 5). During 45° oblique loading, stress was concentrated at the corresponding position of the periodontal membrane on the lingual side of the lower anterior tooth root close to the neck. The MVMS was concentrated on the mandibular first molar, and the stress was 2–4 times than during vertical loading (Fig. 7, Table 6). It was found that during vertical and oblique loading, the MVMS increased with the aggravation of periodontitis, and stress of the periodontal membrane was increased more significantly in moderate to severe periodontitis.

**Table 5:** Peak stress of periodontal membrance during vertical loading (MPa)

Group	47	46	45	44	43	42	41	31	32	33	34	35	36	37
A	1.12	1.04	0.67	1.16	0.49	1.02	1.21	1.35	1.12	0.46	0.97	0.66	0.92	0.93
B	1.22	1.12	0.77	1.19	0.52	1.17	1.41	1.65	1.29	0.48	1.22	0.78	1.28	1.00
C	1.69	1.40	0.86	1.55	0.60	1.43	1.93	2.33	1.79	0.57	1.52	1.00	1.65	1.31
D	2.59	1.76	1.06	2.13	0.76	2.74	2.74	3.15	2.66	0.71	2.47	1.313	2.13	1.91

**Table 6:** Peak stress of periodontal membrance during 45° oblique loading (MPa)

Group	47	46	45	44	43	42	41	31	32	33	34	35	36	37
A	2.24	3.31	2.63	2.58	0.88	1.08	1.67	2.11	1.47	0.87	2.91	2.95	2.60	1.66
B	2.49	3.83	3.19	3.00	1.03	1.26	1.89	2.14	1.66	0.87	3.00	3.32	3.91	1.94
C	5.14	3.64	3.87	3.94	1.38	1.69	2.70	2.81	2.14	1.13	4.04	4.63	5.40	2.83
D	6.30	8.93	5.36	5.27	1.53	2.39	2.86	3.70	3.28	1.38	7.77	7.33	8.06	4.34

### Biomechanical analysis of the periodontal cortical bone interface

**Fig. 8:**
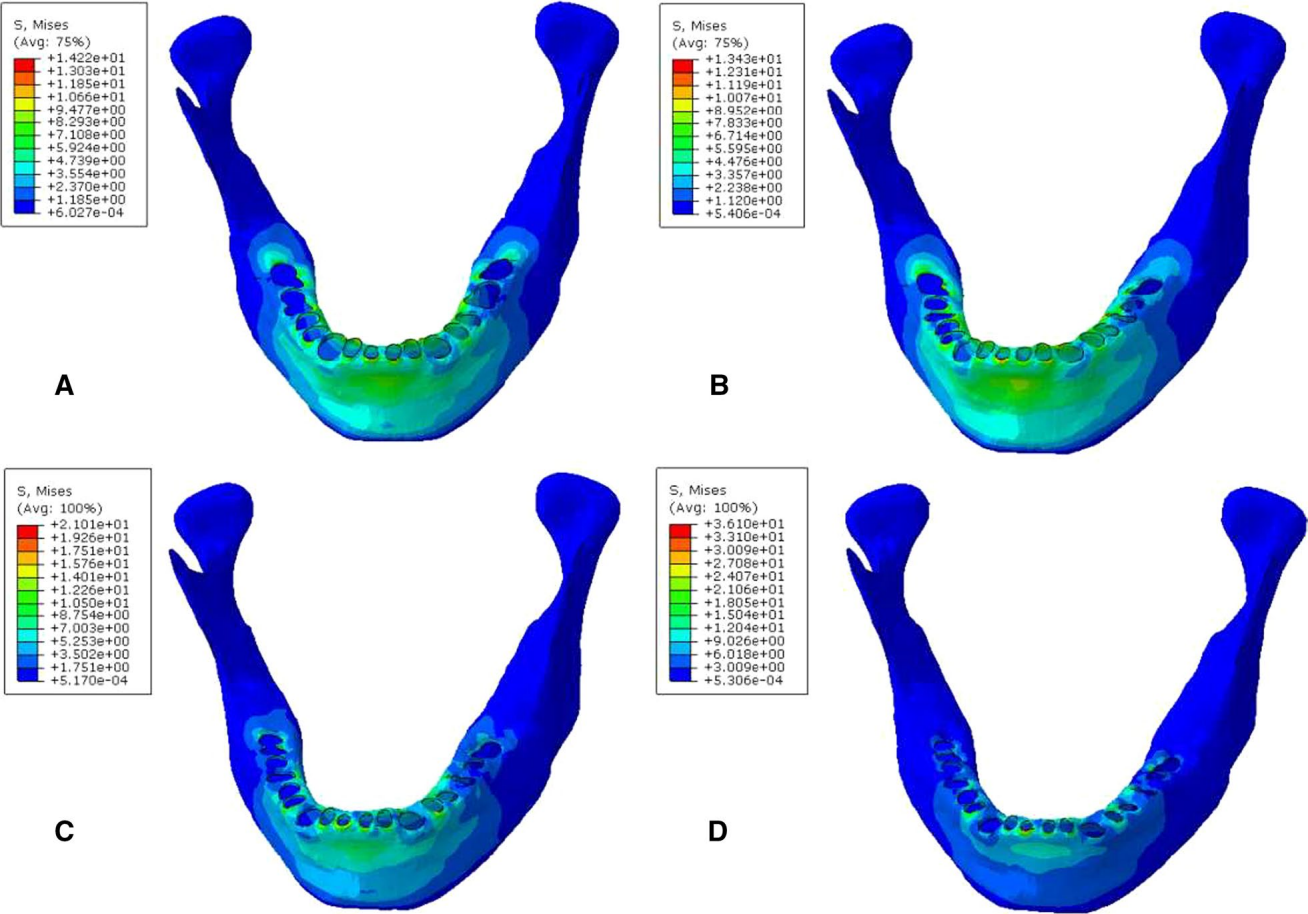
Cloud chart of a finite element analysis of a periodontal cortical bone of mandible during vertical loading. **A** normal mandible; **B** mandible with mild periodontitis; **C** mandible with moderate periodontitis; **D** mandible with severe periodontitis

**Fig. 9:**
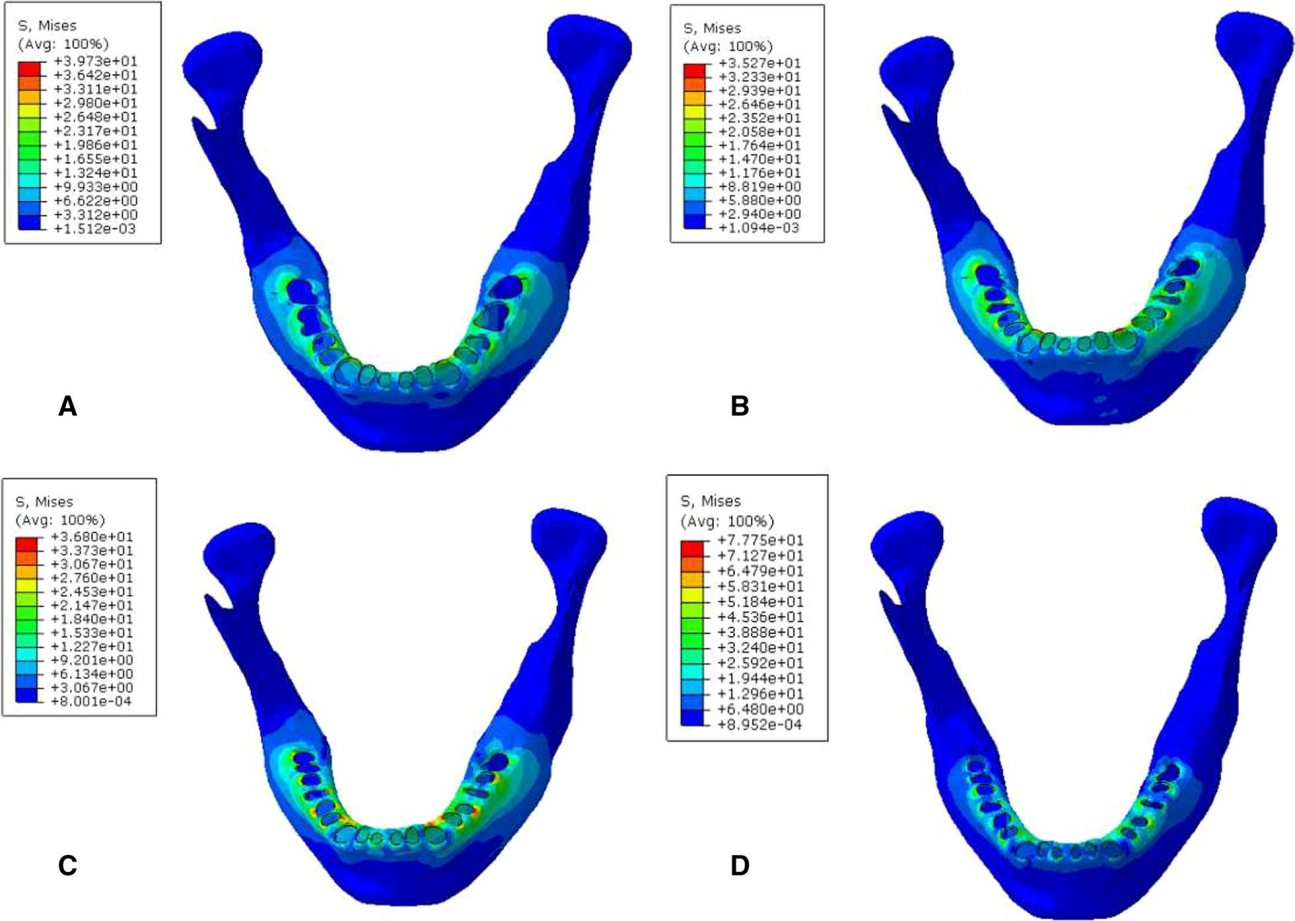
Cloud chart of a finite element analysis of a periodontal cortical bone of mandible during 45° oblique loading. **A** normal mandible; **B** mandible with mild periodontitis; **C** mandible with moderate periodontitis; **D** mandible with severe periodontitis

During vertical loading, occlusal force was transmitted to the mandible via the periodontal membrane of the tooth. When the cortical bone of the mandible was stressed, the maximum Von Mises stress was located in the corresponding cortical bone area of the labial neck of the incisor root; the minimum stress was located in the corresponding cortical bone area of the neck of the canine root (Fig. 8, Table 7). With 45° oblique loading, stress was concentrated in the corresponding cortical bone area of the mesiolingual neck of the posterior tooth (Fig. 9, Table 8). It was found that during vertical and oblique loading, the MVMS was increased with aggravation of periodontitis. In mild to moderate periodontitis, stress was increased slowly; in severe periodontitis, cortical bone stress was increased more significantly.

**Table 7:** Peak stress of periodontal cortical bone during vertical loading (MPa)

Group	47	46	45	44	43	42	41	31	32	33	34	35	36	37
A	10.71	11.07	8.241	10.63	8.82	9.90	12.74	14.22	10.03	8.23	8.61	10.21	12.43	10.29
B	11.31	9.92	8.822	9.26	9.45	9.55	11.85	9.49	10.62	9.21	8.56	8.71	13.43	9.53
C	9.44	13.99	8.399	13.08	9.35	15.03	21.01	15.32	12.55	10.69	15.37	9.40	12.19	8.57
D	16.12	16.41	10.09	21.15	11.28	18.18	36.1	31.30	20.02	11.11	16.21	13.25	18.78	32.52

**Table 8:** Peak stress of periodontal cortical bone during 45° oblique loading (MPa)

Group	47	46	45	44	43	42	41	31	32	33	34	35	36	37
A	23.35	26.53	34.96	35.54	25.31	16.09	14.48	16.14	19.03	31.04	39.73	32.86	28.48	24.42
B	26.99	31.82	31.56	35.27	31.56	29.12	13.10	14.90	15.87	34.77	33.07	27.20	25.41	19.42
C	33.02	36.05	36.80	35.67	28.65	13.46	27.35	17.98	17.28	33.40	36.46	36.09	33.44	27.85
D	38.01	57.69	53.92	56.81	37.14	21.46	44.1	38.68	23.41	33.02	58.43	60.99	55.63	77.75

### Biomechanical analysis of the periodontal cancellous bone interface

**Fig. 10:**
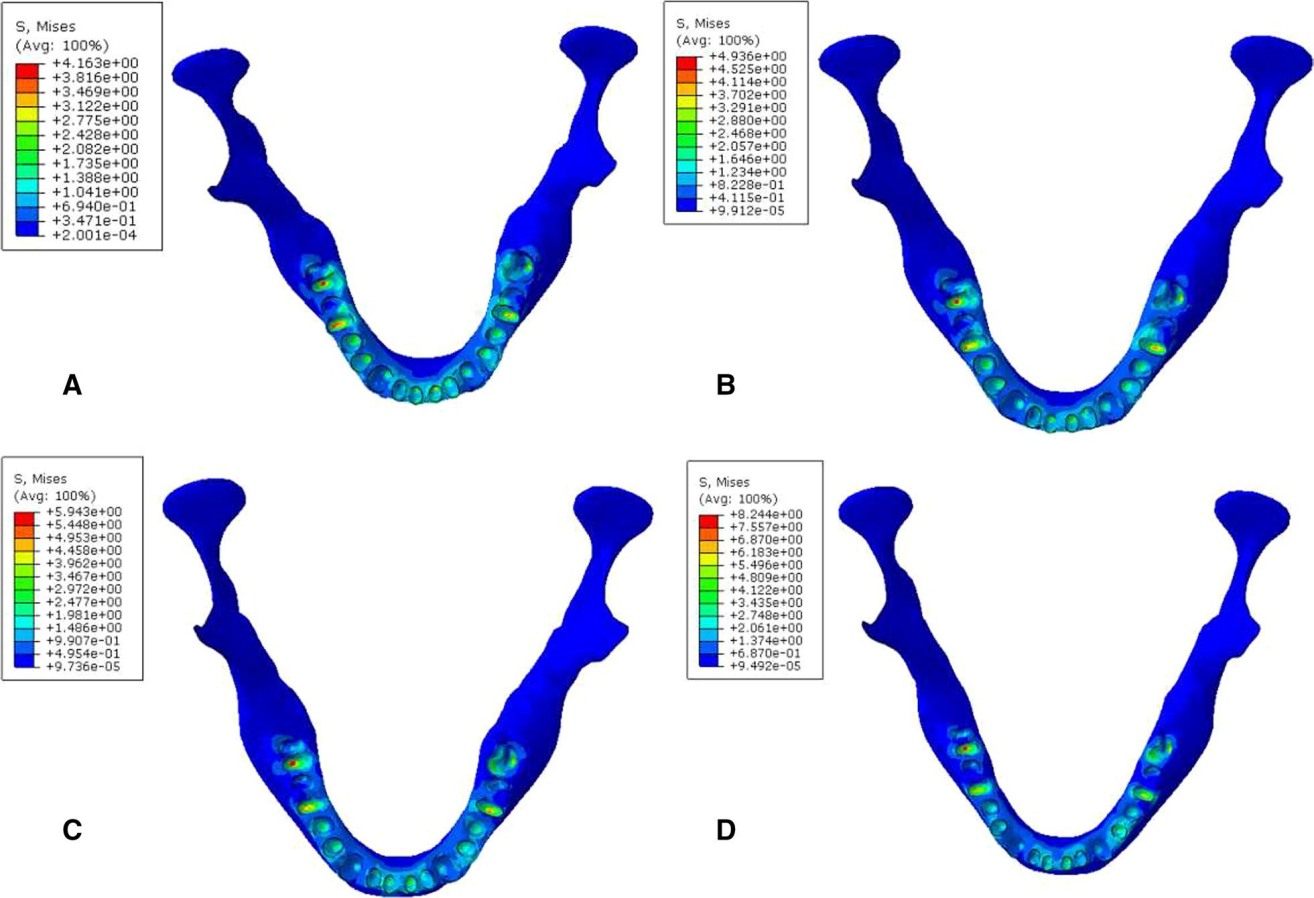
Cloud chart of a finite element analysis of a cancellous bone of mandible during vertical. **A** normal mandible; **B** mandible with mild periodontitis; **C** mandible with moderate periodontitis; **D** mandible with severe periodontitis

**Fig. 11:**
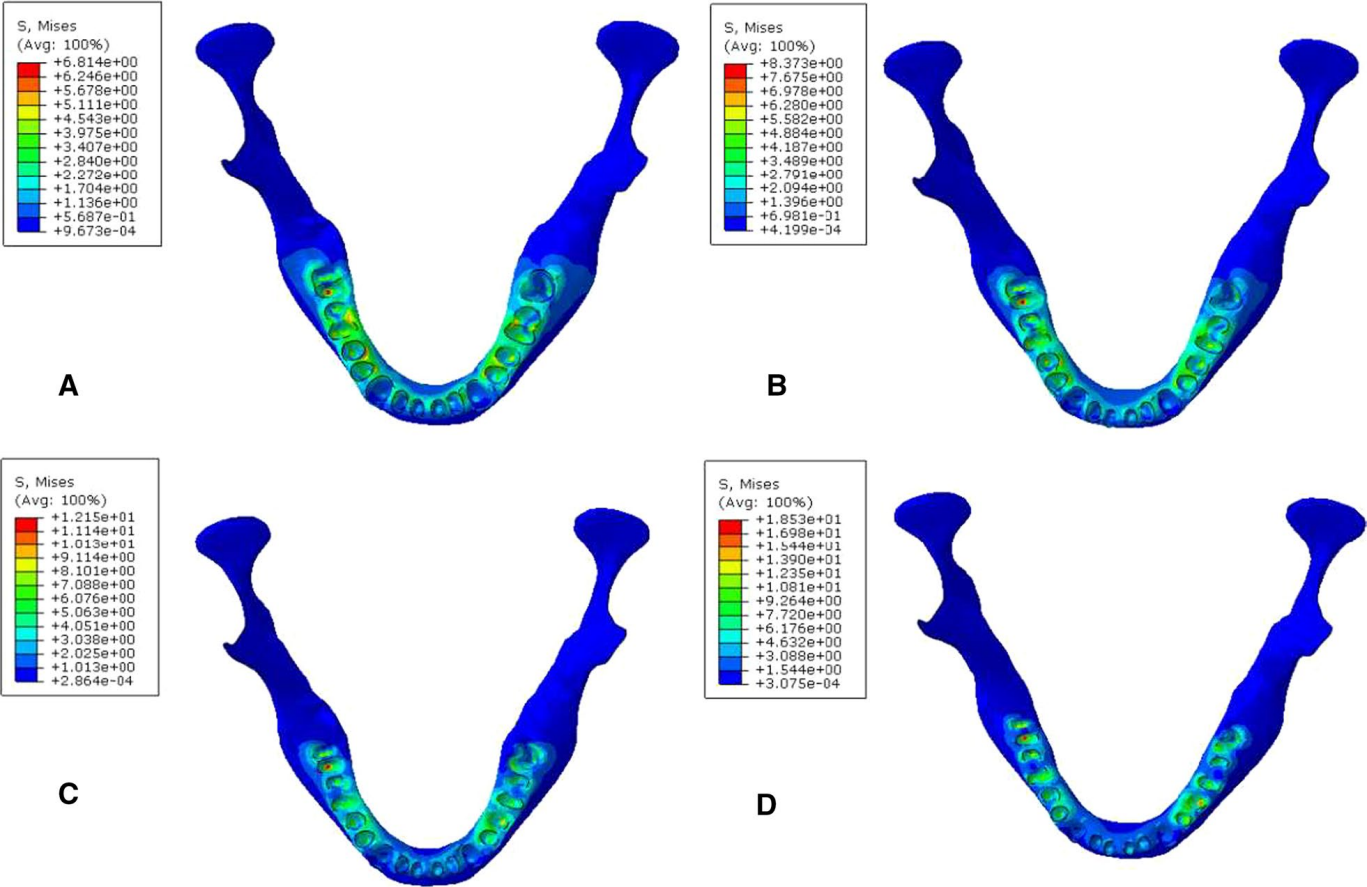
Cloud chart of a finite element analysis of a cancellous bone of mandible during 45° oblique loading. **A** normal mandible; **B** mandible with mild periodontitis; **C** mandible with moderate periodontitis; **D** mandible with severe periodontitis

When the cancellous bone of the mandible was stressed, the stress was mainly distributed in the corresponding cancellous area of the root apex. During vertical and oblique loading, the MVMS was increased with aggravation of periodontitis. The maximum Von Mises stress was located in the apical area of the second molar. In mild to moderate periodontitis, stress increased slowly. In severe periodontitis, stress of cancellous bone increased more significantly (Figs. 10, 11, Tables 9, 10).

**Table 9:** Peak stress of periodontal cancellous bone during vertical loading (MPa)

Group	47	46	45	44	43	42	41	31	32	33	34	35	36	37
A	4.16	3.96	2.31	2.27	1.94	2.27	2.65	3.26	1.78	1.99	1.90	2.19	3.79	3.34
B	4.94	4.66	2.63	2.38	2.27	2.06	3.13	2.98	2.63	1.95	2.10	2.08	4.28	4.40
C	5.94	4.96	2.50	2.53	1.95	2.61	4.10	3.80	2.54	1.85	2.70	2.37	5.48	4.33
D	8.24	5.78	2.95	3.13	2.30	3.53	5.17	5.34	3.95	2.16	4.33	3.10	6.21	6.84

**Table 10:** Peak stress of periodontal cancellous bone during 45° oblique loading (MPa)

Group	47	46	45	44	43	42	41	31	32	33	34	35	36	37
A	6.80	5.53	6.33	6.39	3.64	3.35	2.84	3.46	3.78	3.09	6.81	5.66	5.88	3.36
B	8.37	6.07	6.38	6.88	3.26	2.51	2.76	2.94	3.38	3.18	7.37	6.51	5.84	5.26
C	12.15	7.95	7.64	6.55	3.25	2.91	3.52	4.05	3.22	3.38	6.33	10.22	7.97	8.95
D	18.53	13.54	11.97	12.93	3.36	5.05	4.96	9.24	5.28	3.56	12.53	17.77	11.57	12.41

## Discussions

1. According to experimental results and Tables three to 10, the stress of each structure of periodontal tissue was increased with the degree of alveolar bone resorption. The change in stress of the periodontal membrane of the mandible was similar to the trend of change in the stress of the root surface. In moderate to severe periodontitis, stress was increased significantly, while the stress distribution of cortical bone and cancellous bone of mandible were similar. In severe periodontitis, stress increased significantly. Although the load was within the limit of the physiological tolerance of each tissue, stress fatigue would occur if a repeated load exceeded a certain limit. This result was consistent with alveolar bone resorption and tooth loosening in advanced periodontitis, and the influence of oblique force on stress distribution was greater than that of vertical force [18]. Reddy used the finite element method to analyze changes in the stress of the periodontal membrane of single maxillary incisor at different levels of alveolar bone and found that with decrease in the height of alveolar bone, stress concentration of tooth cusp increased significantly [19]. Some scholars found the maximum tensile stress of the periodontal membrane of a maxillary incisor in the lingual neck area increased with decrease in bone height, but stress decreased with the increase in width of the periodontal membrane, and changes in the height of alveolar bone had little effect on the cortical bone [20]. Based on previous studies, combined with our study, the stress distribution of the labial cortical bone and root in the anterior tooth area of the mandible were concentrated, and the increasing trend was significant with the absorption of alveolar bone. We found that more studies on splinting fixation of the lower anterior teeth were conducted in vivo and in vitro experiments than molars, which was consistent with clinical application. The finite element method is not only of great significance for study of periodontal tissue stress and deformation under loads, it also provides medical and biomechanical experts with a basis for their biomechanical behavior in oral treatment [21].
2. With alveolar bone resorption, the clinical crown is lengthened, which increases the axial force of the root surface. According to the results of finite element analysis, stress increase during oblique loading is more obvious than during vertical loading. Lateral force shall be minimized. Many scholars have used loosened tooth fixation to disperse the occlusal force of lower anterior teeth. Nikolaus used the finite element method to make stress analysis on the therapeutic effects of periodontal splints made of different materials [22], and many scholars have also made clinical evaluation on the treatment timing of loosened tooth fixation [23, 24]. Factors such as wear of occlusal surface and tooth movement in patients with periodontitis will affect the distribution of occlusal force and occlusal contact [25]. However, many studies have directly observed intraoral periodontal parameters for comparative analysis. In combination with study results, for the root surface and periodontal membrane interface, stress increase is particularly significant in moderate to severe periodontitis. In order to ensure uniform stress distribution and reduce stress concentration, and prevent lower anterior teeth from loosening and falling out, patients with moderate to severe periodontitis may undergo preventive internal fixation of lower anterior teeth to disperse occlusal force.
3. The mandible model of periodontitis in the study simulates bone level through alveolar ridge trimming. The mandibular alveolar ridge at the defect site has no obvious structural change except for loss of height. Under actual situations, alveolar bone resorption in periodontitis is not uniform and continuous horizontal resorption, and the depth of periodontal pockets on different sides of each tooth is not the same. According to literature reports, there are differences in the morphology and characteristics of a normal mandible and alveolar ridge trophic mandible, and bone characteristics and evolution, as well as changes in bone density shall also be taken into account for mandible changes [26, 27]. With rapid development of computer medical imaging technology and medical technology, application of the finite element method in the field of stomatology can achieve more accurate biomechanical analysis in vitro for various oral diseases, thereby simulating and guiding clinical practice [22].
  Many scholars [4, 28] assessed 162-227 splinted teeth, splint survival and number of plint-repairs retrospectively over 10 years to indicated that splinting can assist the retention of periodontally affected teeth by reducing their mobility. The significance of biomechanical analysis of mandibular models with varying degrees of alveolar bone resorption was to conduct mechanical analysis, and evaluation of the tooth and periodontal tissues of patients with periodontitis, to avoid stress concentration, retard bone absorption and then to prompt clinical optimization treatments, improve long-term survival rate of teeth.
4. Finite element analysis has some limitations that should be considered. Contact boundaries, isometric characters of materials, and static loading jeopardize the validity of analysis. Nevertheless, the simplifications adopted in this study are not expected to fundamentally alter the behavior of bone materials, and therefore, this analysis should help advance a better understanding periodontal changes of alveolar bone resorption.
5. As for future studies, to validate the results obtained in this simulation, further clinical and longitudinal follow- up studies are required. And it should be detail the biological behavior of all the materials to build vitro models closer to the clinic.

## Conclusion

Teeth with moderate to severe periodontitis, loosened tooth fixation can be used to retard bone absorption based on the Finite Element Analysis, especially for lower anterior teeth.

## Data Availability

All data generated or analyzed during this study are included in this published article [and its supplementary information files].
